# Reinforcement Effects of Shear Thickening Fluid over Mechanical Properties of Nonwoven Fabrics

**DOI:** 10.3390/polym14224816

**Published:** 2022-11-09

**Authors:** Chen-Hung Huang, Chih-Hua Chien, Bing-Chiuan Shiu, Yueh-Sheng Chen, Jia-Horng Lin, Ching-Wen Lou

**Affiliations:** 1 Department of Aerospace and Systems Engineering, Feng Chia University, Taichung 407102, Taiwan; 2 Laboratory of Fiber Application and Manufacturing, Department of Fiber and Composite Materials, Feng Chia University, Taichung 407102, Taiwan; 3 Fujian Key Laboratory of Novel Functional Textile Fibers and Materials, Minjiang University, Fuzhou 350108, China; 4 Department of Biomedical Engineering, China Medical University, Taichung 404333, Taiwan; 5 Department of Bioinformatics and Medical Engineering, Asia University, Taichung 413305, Taiwan; 6 School of Chinese Medicine, China Medical University, Taichung 404333, Taiwan; 7 Advanced Medical Care and Protection Technology Research Center, College of Textile and Clothing, Qingdao University, Shandong 266071, China; 8 Innovation Platform of Intelligent and Energy–Saving Textiles, School of Textile Science and Engineering, Tiangong University, Tianjin 300387, China; 9 Department of Medical Research, China Medical University Hospital, China Medical University, Taichung 404333, Taiwan

**Keywords:** shear thickening fluid, polyethylene glycol, silicon dioxide, nonwoven fabrics, dynamic impact

## Abstract

Conventional personal protective equipment is usually made in multilayer stacks, and appears clumsy and uncomfortable, offering limited protection. In recent years, a newly-developed nanosuspension, shear thickening fluids (STFs), has been commonly applied to buffer and shock absorption. In this study, nonwoven fabrics are impregnated with 30 wt%, 35 wt%, or 40 wt% STF in order to strengthen the interaction among fibers. The resultant STF composite nonwoven fabrics are observed for their morphology, and tested for their tensile strength, tearing strength, bursting strength, and dynamic impact resistance, thereby examining the damage resistance of the materials. The SEM images indicate that the fibers are adhered with a tremendous amount of silicon dioxide (SiO_2_) particulates with a rise in the STF concentration, due to which the smooth fibers become rough. Moreover, the mechanical test results indicate that a rise in the STF concentration improves the frictional force during the relative motion of fibers, which subsequently mechanically strengthens the STF composite nonwoven fabrics. The dynamic impact test results show that when the STF concentration increases from 30 wt% to 35 wt%, the materials exhibit dynamic impact strength that is significantly improved to 51.9%. Nonetheless, significant improvement in dynamic impact strength is absent when the STF concentration increases to 40 wt%. To sum up, a critical value of STF concentration has a positive influence over the mechanical strengths of STF composite nonwoven fabrics.

## 1. Introduction

Public discourse has recently emphasized protection and safety. Many countries restrict and regulate the use of guns, but overlook the threat of sharp tools, e.g., knives, which are everywhere. Among the diversity of industrial fields, employees in the machining field are frequently in jeopardy due to a shortage of caution, to compensate for which the use of personal protective equipment offers timely protection. With regard to traditional protective equipment that consists of heavy steel irons or multi-layered textiles, the disadvantages include a heavy weight, lower comfort, and loss of mobility [1,2]. In recent years, scientists have been devoted to developing light-weight and flexible protection gear, shear thickening fluid (STF), a non-Newtonian liquid, has been commonly used in studies on buffer and shock absorption [3,4,5,6,7]. At a micron or nanometer scale, STF particulates are distributed in a dispersed medium, forming a highly-concentrated solid–liquid colloidal suspension [8,9]. STF exhibits a reaction mechanism. When at a normal status, STF appears to be a thick liquid with mobility. When receiving an exterior impact force that exceeds its critical force, the STF particulates demonstrate collision and aggregation, transforming from liquid to solid in order to adsorb the impact energy, during which the structure of the STF becomes robustly stable, with buffer and shock absorption. As soon as the impact force disappears, the status of STF reverses from solid to liquid (its original state) [10,11,12].

To improve the flexibility of protection gear, Khodadadi et al. and Wagner et al. incorporated STF into Kevlar fabrics, and the composites were evaluated with ballistic and puncture resistance tests [13]. The test results indicated that with STF finishing, Kevlar fabrics exhibited strengthened bulletproof performances. Moreover, 4-layered STF/Kevlar composites demonstrated bulletproof performance as good as that of 14-layered pure Kevlar fabrics. Meanwhile, STF/Kevlar composites exhibited flexibility that was superior to that of pure Kevlar fabrics [14]. Furthermore, the rheological properties of STF played an important role in the mechanical reinforcement of fabrics. Although the energy absorption of STF is in direct proportion to the load of silicon dioxide (SiO_2_) nanoparticles, an excessive load of SiO_2_ nanoparticles adversely affects the STF effectiveness [15]. Furthermore, Majumdar et al. compared the Kevlar woven fabrics that were and were not processed with STF in terms of the deformation and energy absorption modes. The control group (without STF) only allowed the primary yarns of the Kevlar woven fabrics that were in direct contact with the impactor to participate in the impact resistant reaction; meanwhile, the energy absorption of the woven fabrics was compromised due to the slippage of yarns. In contrast, the STF-containing fabrics consisted of STF that served as a bridge that joined all yarns in proximity, thus the secondary yarns surrounding the primary yarns were also involved in the impact resistance reaction (i.e., energy absorption). It was clear that the experimental group demonstrated the structural failure due to yarn rupture, instead of yarn slippage [16]. The presence of STF prevents the yarn from slipping while strengthening the impact resistance of fabric, as demonstrated by Cao et al. [17].

Haris et al. examined how an STF system influenced the ballistic penetration of STF/Kevlar composite materials. They concluded that with particulates that reached a certain level of volume fraction, composites were able to embody the ballistic penetration [18]. According to the low-velocity impact test, Mawkhlieng et al. confirmed that after Kevlar fabrics were treated with monodispersed STF, the incorporation of STF considerably strengthened the Kevlar fabrics in terms of energy absorption and peak force [19]. Furthermore, Xu et al. and Gürge et al. proved that a high concentration of STF and a large particle size of silicon dioxide (SiO_2_) nanoparticles demonstrated a positive influence on the puncture resistance of STF-containing composites [20,21]. Additionally, the rheological properties of STF are also dependent on the particulate size, particulate type, particulate volume fraction, preparation method, temperature, and dispersed medium [19,22,23,24]. Gunjan et al. studied the relationship between the impact resistance of STF composites and the parameters (particle size, concentration, and rheological parameter). The viscosity of STF was in direct proportion to the SiO_2_ particle size. The greater the particulate size, the higher the frictional force among particulates, which can easily counteract the impact energy caused by the externally applied impact force [25]. Moreover, Wei et al. investigated how yarn slippage was correlated with impact resistance and energy absorption. They found that yarns had a relative interface sliding ratio that was dependent on the number of layers, fabric structure, and laying angle, and subsequently the yarn sliding ratio affected the rheological state of STF as well as the impact resistance of fabrics [26].

To sum up, the incorporation of STF mainly reduces the slippage of fibers and yarns [27]. STF can effectively strengthen the impact resistance of woven and knitted structures, but STF is rarely used in the nonwoven field. Hence, in this study, 30 wt%, 35 wt%, and 40 wt% of SiO_2_ particulates are combined with polyethylene glycol (PEG 200), forming shear thickening fluids (STFs) at different concentrations. STFs are then combined with nonwoven fabrics to build STF-containing composites, thereby enhancing the interaction among fibers. Next, the morphology of nonwoven fabrics is observed, after which tensile strength, tearing strength, bursting strength, and dynamic impact measurements are conducted to measure the mechanical properties of STF composites, thereby examining the influence of STF impregnation at different concentrations.

## 2. Experimentals

### 2.1. Material

Recycled Nomex woven selvedges (FORMOSA Taffeta Co., Ltd., Yunlin, Taiwan) have a molecular structure consisting of a benzene ring that has stiffness and thus can endure high temperatures. Fire-retardant polyester fibers (Far Eastern New Century Corporation, Taipei, Taiwan) have specifications of 6D × 64 mm, and they are formed as a result of a condensation reaction among saturated dibasic acid, dibasic alcohol, and phosphorus series, demonstrating fire-retardant effects. Low-melting-point polyester (LMPET) fibers (Huvis Company, Seoul, Korea) have specifications of 4D × 51 mm. A high temperature can soften the surface layers of LMPET fibers without changing the structure of the core layer, thereby thermally bonding the nonwoven fabrics while reinforcing the mechanical properties. STF is prepared with silicon dioxide (SiO_2_) particulates (CHOKO Co., Ltd., Kaohsiung, Taiwan) and polyethylene glycol (PEG) (First Chemical Manufacture Co., Ltd., Taipei, Taiwan). SiO_2_ particulates have an average particle size smaller than 100 nm and a specific surface area greater than 500 m^2^/g. PEG has a molecular weight of 200. PEG is toxicity free and compatible with diverse solutions, which makes it a qualified dispersed medium.

### 2.2. Preparation of Samples

#### 2.2.1. Nonwoven Fabrics

**Figure 1:**
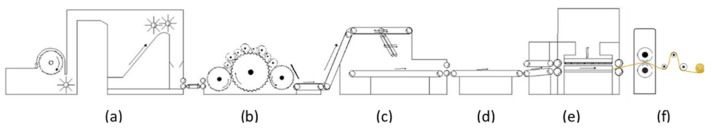
Manufacturing process diagram for nonwoven fabrics that involves with (**a**) the hopper feeder; (**b**) carding machine; (**c**) Folding machine; (**d**) transmission band; (**e**) needle punching machine; (**f**) Drum heat press.

A nonwoven manufacturing process was employed to form the matrices. The recycled Nomex woven selvages were crumbled and then mixed with fire-retardant polyester fibers and low-melting-point polyester fibers, as shown in Figure 1a. The mixed staple fibers were combed with a carding machine in order to form aligned fiber webs, as shown in Figure 1b. The webs were laminated using a folding machine, as shown in Figure 1c, thereby forming multi-layered webs. Next, a transmission band (Figure 1d) was used to deliver the webs to a needle-punching machine (Figure 1e) to form needle-punched nonwoven matrices. To acquire better mechanical properties, nonwoven matrices were hot pressed with a hot-pressing machine, as shown in Figure 1f, which melted the sheath of the low-melting-point polyester fibers, which subsequently formed thermally-bonded points that bonded the constituent fibers, providing the nonwoven matrices with greater mechanical performance [28]. Table 1 shows the specifications of the nonwoven fabrics.

#### 2.2.2. Preparation of Composite Nonwoven Fabrics

**Figure 2:**
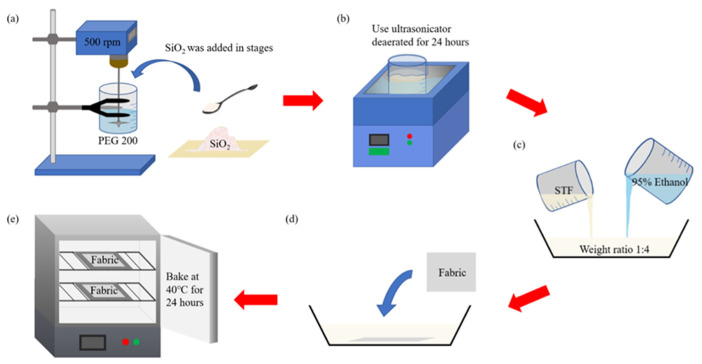
The preparation process of STF composites: (**a**) formulation of STF; (**b**) de-aerating STF; (**c**) dilution of STF; (**d**) STF impregnation of nonwoven fabrics; (**e**) thermal treatment of nonwoven fabrics.

STF was formulated with PEG and SiO_2_ particulates at a ratio of 30 wt%, 35 wt%, and 40 wt%. Next, STFs were combined with nonwoven composites, as shown in Figure 2. As SiO_2_ particulates have a high volume per unit weight, with a small proportion of liquid and a greater proportion of solids, SiO_2_ particulates and PEG cannot be well mixed in one instance. A high-torque mixer (Figure 2a) was thus used to mix PEG and a small amount of SiO_2_ particulates [29], after which the beaker was sealed and then underwent 24 h de-aeration via an ultra-sonicator, as shown in Figure 2b, thereby formulating STFs. As a result, the STF exhibited a considerable viscosity, which meant that the STF-impregnation required dilution with ethanol (STF/ethanol: 1/3) before nonwoven fabrics were immersed, as shown in Figure 2 [30]. The diluted STF was evenly distributed with an ultrasonicator, as shown in Figure 1d, after which nonwoven fabrics underwent STF impregnation and were then dried at 40 °C for 24 h in a baking oven. Finally, samples were removed and trimmed as specified in the standard tests.

### 2.3. Measurements

#### 2.3.1. Morphological Characterization

A scanning electron microscope (SEM, HITACHI S-4800, Tokyo, Japan) was employed to observe the microstructure of samples surface. Samples were dried in advance for 24 h at 40 °C in a baking oven. Next, carbon tape was used to fix the sample over the custom-made baseplate of SEM. Samples were coated with a thin layer of gold.

#### 2.3.2. Tensile Strength Test

A universal testing machine (HT-2402, Hung Ta Instrument Co., Ltd., Taichung, Taiwan) was used to measure the tensile strength at a test rate of 305 mm/min as specified in D5035-11. Samples had a size of 180 mm × 25.4 mm. The distance between the upper and lower fixtures was 75 mm. Ten samples for each specification were taken both along the machine direction (MD) and the cross-machine direction (CD), and as such provided the average tensile strength [31].

#### 2.3.3. Tearing Strength Test

As specified in the ASTM D4533-11 test standard, a universal testing machine (HT-2402, Hung Ta Instrument Co., Ltd., Taichung, Taiwan) was used to measure the tearing strength of samples at a constant tensile speed of 300 mm/min. The trapezoid samples had a size of 150 mm × 75 mm. The long base of the trapezoid was prepared with a 15 mm cutting. Ten samples for each specification were taken along both the machine direction (MD) and the cross-machine direction (CD), and as such provided the average tearing strength.

#### 2.3.4. Bursting Strength Test

**Figure 3:**
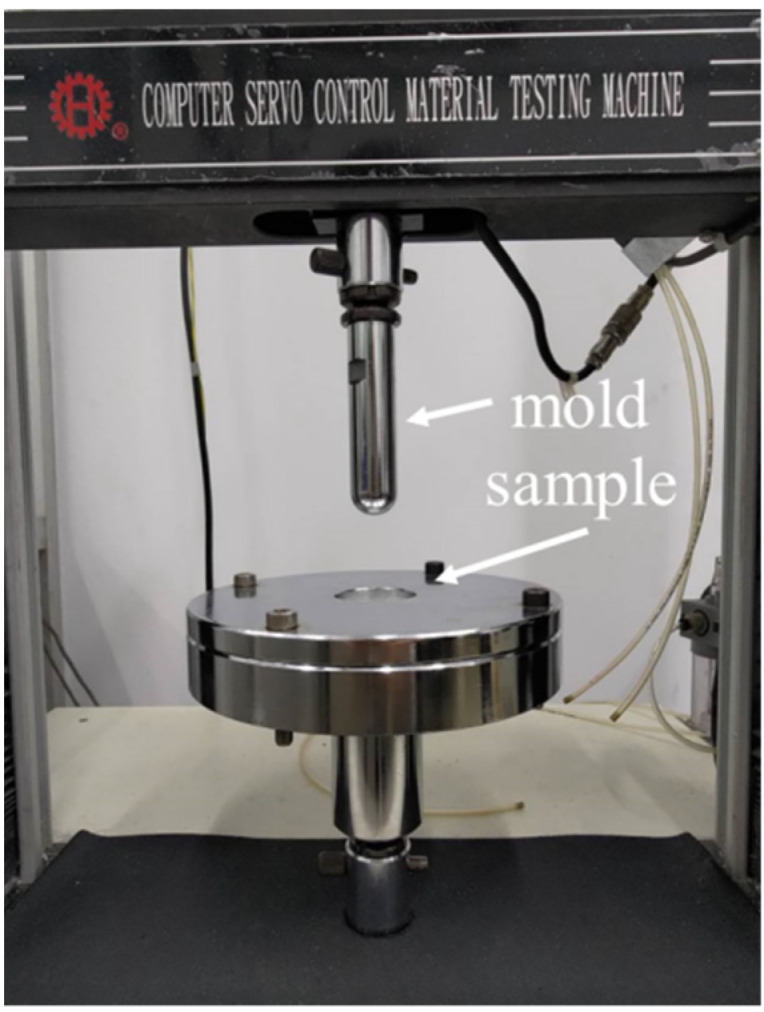
The assembly of bursting strength test.

As specified in the ASTM D3786 test standard, a universal testing machine (HT-2402, Hung Ta Instrument Co., Ltd., Taichung, Taiwan) was used to measure the tearing strength of samples at a test rate of 100 mm/min. Samples had a size of 100 mm × 100 mm. Ten samples for each specification were used for the average. Figure 3 shows the assembly, where the sample is mounted in the upper and lower molds, and a semicircular die with a diameter of 25 mm was used to burst the sample. The maximal bursting strength is recorded in order to compute the average [32].

#### 2.3.5. Dynamic Impact Test

**Figure 4:**
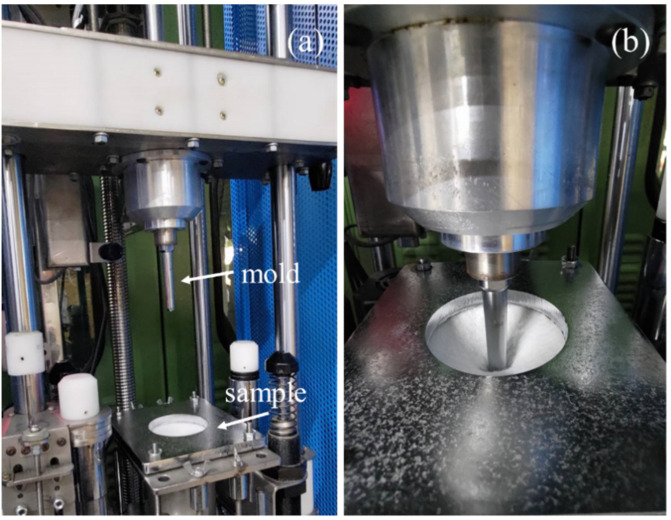
(**a**) Schem of dynamic impact strength test; (**b**) Test the situation.

A drop weight impact tester (KNM-FC0701, Changfata Industrial Co., Ltd., Taichung, Taiwan) was used to measure the dynamic impact strength, as specified in the ASTM D7192, and shown in Figure 4a. Samples had a size of 100 mm × 100 mm and five samples for each specification were used for the test. A sample was fixed over the platform and the mold was lifted up to a height that was 300 ± 10 mm away from the sample. The mold, weighing 8.5 kg, was released in free fall in order to strike the sample with a total impact energy of 25 J. The mold was bullet-shaped and had a diameter of 12.7 mm. When the mold penetrated the sample, as shown in Figure 4b, the maximal dynamic impact strength (N) was yielded.

## 3. Results and Discussion

### 3.1. Morphological Characterization

**Figure 5:**
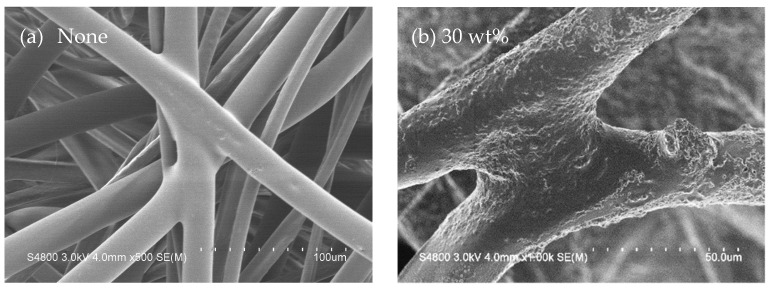

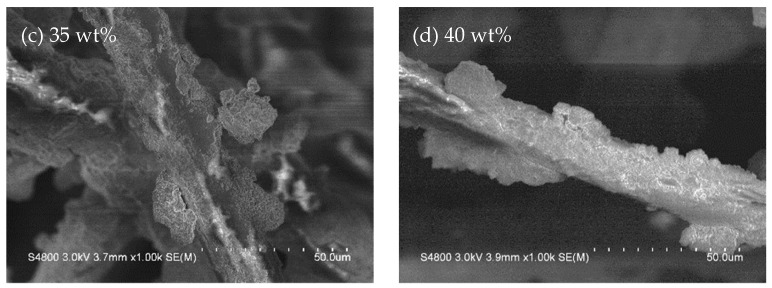
SEM images of (**a**) control group; and STF-saturated composite nonwoven fabrics as related to the STF concentration, being: (**b**) 30 wt%.; (**c**) 35 wt%.; (**d**) 40 wt%.

Figure 5 shows the SEM images of nonwoven fabrics as related to STF impregnation. Figure 5a shows that the fibers are connected with hot bonding points as a result of hot-pressing temperature. Figure 5b shows that with an STF concentration of 30 wt%, SiO_2_ particulates are distributed over the fibers and the particulate size is much smaller than the fiber diameter. Based on Figure 5c,d, due to a rise in the STF concentration and a large specific surface area of SiO_2_ particulates, there are more SiO_2_ particulates adhering to the nonwoven fabrics, especially at the STF concentration of 40 wt%, which enables the whole fiber to be wrapped in SiO_2_ particulates. The smooth surface of fibers becomes rough, which in turn affects the performances of the resulting nonwoven fabrics. In addition, the white bright areas in Figure 5c,d are attributed to the aggregation of SiO_2_ particulates, and the surface becomes rough, concave, and convex. Therefore, electrons are accumulated over the concave parts, and then reflect the white light during the measurement.

### 3.2. Tensile Strength of STF Composite Nonwoven Fabrics

**Figure 6:**
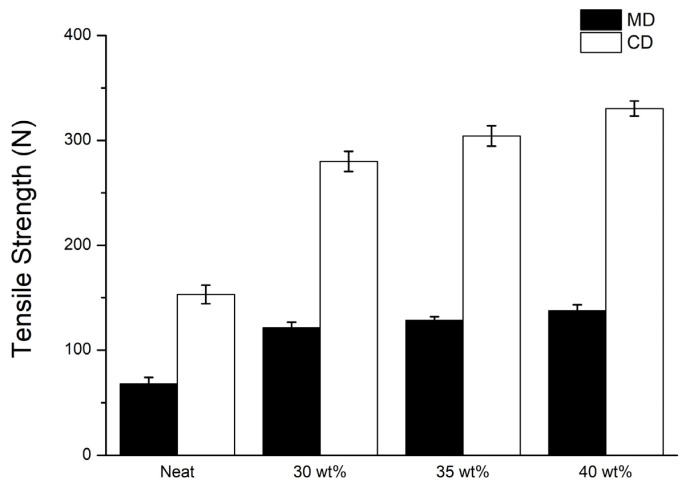
Tensile strength of STF composite nonwoven fabrics as related to the STF concentration.

Figure 6 and Table 2 shows the tensile strength of STF composite nonwoven fabrics as related to the STF concentration. As for the control group (i.e., non-STF saturated), tensile strength along the machine direction (MD) is 67.9N, and along the cross-machine direction (CD) is 153.0 N. Moreover, a rise in the concentration of STF impregnation has a positive influence on the tensile strength of STF composite nonwoven fabrics regardless of whether it is along the MD or CD. The tensile strength along the MD is improved by 78.6%, 89.3%, and 102.4% when the STF concentration is 30 wt%, 35 wt%, and 40 wt%, respectively. Furthermore, the tensile strength along the CD is 83.1%, 98.8%, and 115.9% when the STF concentration is 30 wt%, 35 wt%, and 40 wt%, respectively. The results are highly associated with the SiO_2_ particulates over the fibers. During the tensile strength test, a greater STF concentration contributes to a higher surface roughness of the fibers due to the adhesion of SiO_2_ particulates, thereby providing a greater frictional force among fibers and thus improving tensile strength. The results are in conformity with the finding of the studies by Majumdar et al. and Cao et al [16,17].

### 3.3. Tearing Strength of STF Composite Nonwoven Fabrics

**Figure 7:**
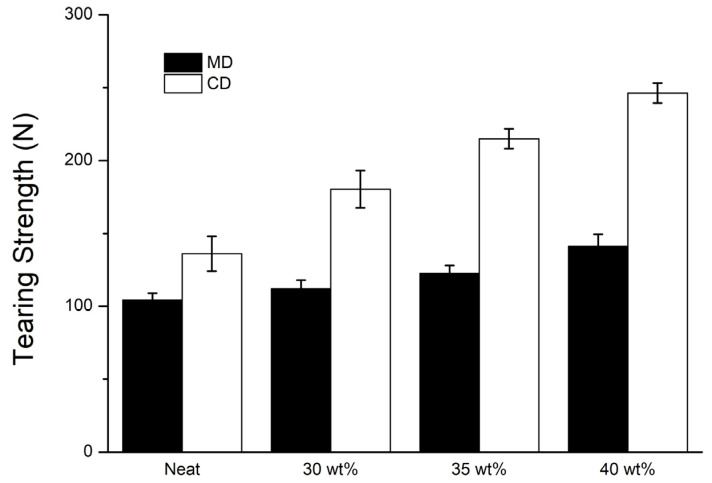
Tearing strength of STF composite nonwoven fabrics as related to the STF concentration.

Figure 7 and Table 2 shows the tearing strength of STF composite nonwoven fabrics as related to the STF concentration. As for the control group, the non-impregnation nonwoven fabrics have a tearing strength along the MD of 104.3, and along the CD of 136.1N. The STF impregnation demonstrates a significant influence on the tearing strength; in particular, where the STF concentration is 40 wt%, the tearing strength along the CD is improved to 81.0%. The reinforcement in the tearing strength of STF composite nonwoven fabrics is ascribed to the presence of SiO_2_ particulates, which strengthen the frictional force that confines the fiber slippage during the tearing strength measurement. By contrast, the tearing strength of STF composite nonwoven fabrics along the MD is not as high as along the CD. The tearing strength along the MD is only 7.6%, 17.6%, and 35.5% when the nonwoven fabrics are processed with 30 wt%, 35 wt%, and 40 wt% STF-impregnation, respectively. It is surmised that the tearing strength of nonwoven fabrics along the MD is the same direction as the fiber alignment. Namely, the constituent fibers demonstrate lower friction force and show an insignificant improvement in the tearing strength.

### 3.4. Bursting Strength of STF Composite Nonwoven Fabrics

**Figure 8:**
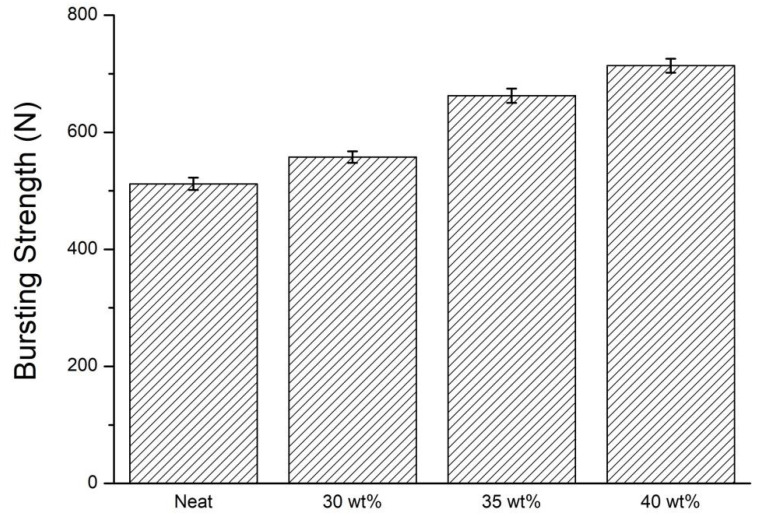
Bursting strength of STF composite nonwoven fabrics as related to the STF concentration.

**Figure 9:**
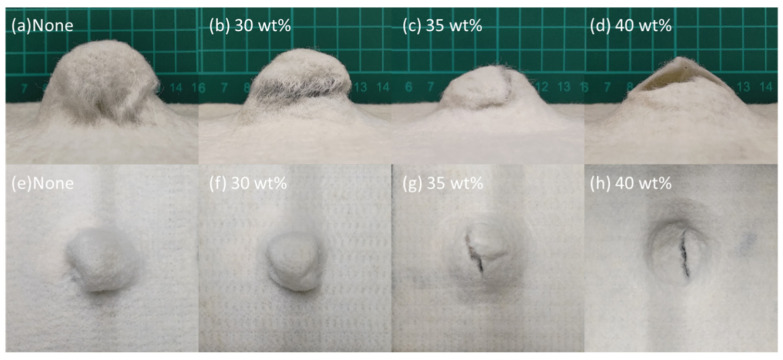
Images of samples after the bursting strength test: (**a–d**) the lateral view; (**e–h**) the top view.

**Figure 10:**
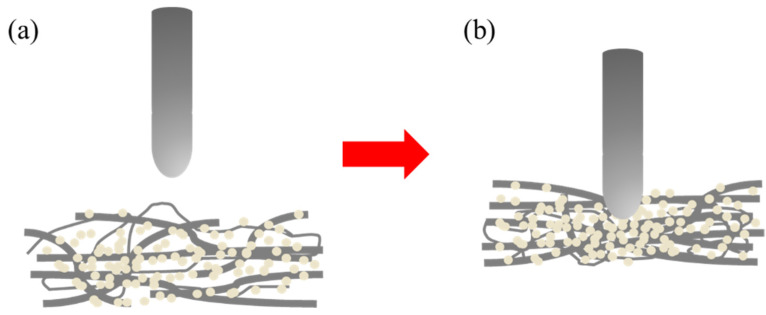
Illustrative diagram of STF composite nonwoven fabrics with (**a**) an even distribution before the test; (**b**) the collision of SiO_2_ particulates during the test.

Figure 8 and Table 3 shows the bursting strength of STF composite nonwoven fabrics. The control group demonstrates a bursting strength of 512.0 N, which only involves the impactor and primary fibers. The structure is destroyed, mainly due to the fiber slippage, which in turn causes a lower energy absorption. Additionally, the fiber slippage is also present when non-treated nonwoven fabrics are exerted with an impact force, as shown in Figure 9a,c. Contrarily, the nonwoven fabrics that are processed with STF impregnation show a bursting strength that is in direct proportion to the STF concentration. The bursting strength—with corresponding STF concentration—is strengthened by 8.9% (30 wt%), 29.4% (35 wt%), and 39.4% (40 wt%). In addition, Figure 9 shows that the damage level is exacerbated with a rise in the STF concentration. When the STF concentration is 30 wt% (Figure 9b,f), there are a proportion of fibers demonstrating slippage, which in turn makes the increase in the bursting strength less significant. With the STF concentration increasing to 35 wt% and 40 wt%, more STF renders the primary fibers (i.e., fibers that impactor strikes) and the secondary fibers (i.e., the remaining fibers) resistant to the impact damage. Figure 10a shows that the SiO_2_ particulates are uniformly distributed over the fiber surface, while Figure 10b shows that SiO_2_ particulates collide and aggregate to respond to the impact damage, which enables nonwoven fabrics to absorb considerable transient impact energy immediately; eventually, the STF nonwoven fabrics show improved bursting strength.

### 3.5. Dynamic Impact Strength of STF Composite Nonwoven Fabrics

**Figure 11:**
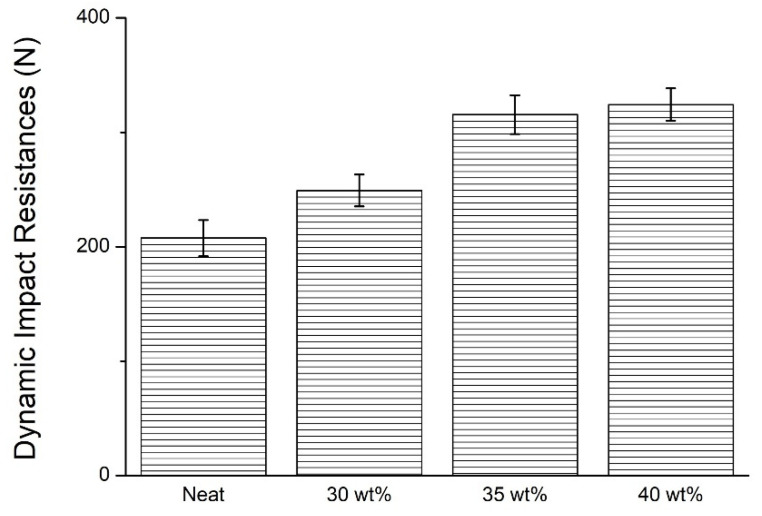
Dynamic impact strength of STF composite nonwoven fabrics as related to the STF concentration.

**Figure 12:**
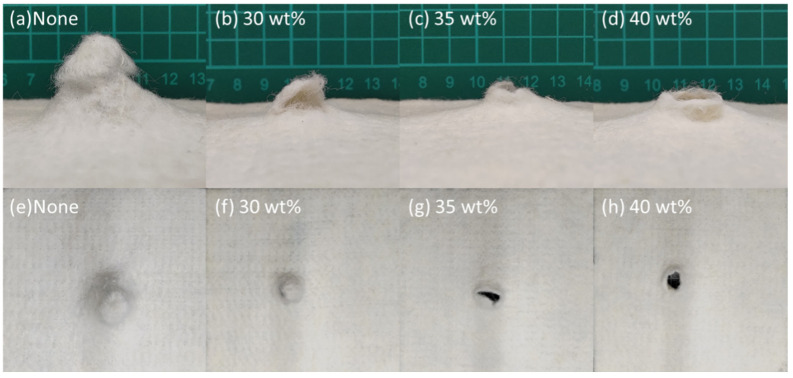
Images of samples after the dynamic impact test: (**a–d**) the lateral view; (**e–h**) the top view.

Figure 11 and Table 3 shows the dynamic impact strength of STF composite nonwoven fabrics as related to the STF concentration. The non–STF impregnated nonwoven fabrics have a dynamic impact strength of 207.7N. When being exerted with the impact, only the primary fibers that are in contact with the impactor react with the impact energy. However, with the incorporation of STF, a greater STF concentration provides the STF composite nonwoven fabrics with greater impact energy absorption capacity. Hence, the dynamic impact strength–with corresponding STF concentration–is 19.9% (30 wt%), 51.9% (35 wt%), and 56.1% (40 wt%). The non-STF treated nonwoven fabrics, as shown in Figure 12a,e, indicate that the majority of fibers are pulled and thus cause slippage to resist an impact force. The STF impregnation strengthens the frictional force among fibers, and the frictional force is in direct proportion to the STF concentration. Namely, a high STF concentration renders the primary fibers and the remaining fibers more able to resist the dynamic impact energy. To sum up, the structural failure is attributed to the breakage of fibers, as shown in Figure 12c,d,g,h, rather than the slippage of fibers.

Furthermore, it is clearly observed that the mechanical properties of nonwoven fabrics are greatly improved when the STF concentration increases from 30 wt% to 35 wt%. However, when the STF concentration increases from 35 wt% to 40 wt%, the improvement level in energy absorption and mechanical reinforcement are lower compared to the previously mentioned STF concentration range. The results show a trend consistent with the findings in the studies by Khodadadi et al. and Gunjan et al. The energy absorption of nonwoven fabrics is in direct proportion to the SiO_2_ nanoparticles loaded in the STFs. When it exceeds the loading critical value, STF no longer strengthens mechanical properties as much as it can, so the mechanical reinforcement is thus found to be gradually reduced [15,25].

## 4. Conclusions

Different weight percentage concentrations of shear thickening fluids (STFs) were formulated to saturate nonwoven fabrics made by hot-pressing Nomex recycled selvages, flame-retardant polyester fibers, and low-melting-point polyester fibers, thereby forming STF composite nonwoven fabrics. Morphology observation, as well as tensile strength, tearing strength, bursting strength, and dynamic impact measurements were conducted to evaluate the mechanical performances of STF composite nonwoven fabrics. SEM images show that a greater STF concentration results in a considerable adhesion amount of SiO_2_ particulates over the fibers, and fibers appear rugged, which suggests that the STF composite nonwoven fabrics are mechanically strengthened because the frictional force is increased. The tensile strength is improved by 102.4% (MD) and 115.9% (CD), while the tearing strength is increased by 35.5% (MD) and 81.0% (CD). Moreover, the bursting strength and dynamic impact strength measurements show that when exerted with an external force, SiO_2_ particulates demonstrate collision, aggregation, and then bonding, which makes the primary fibers and the secondary fibers in proximity resist the force concurrently. As a result, the bursting strength and the dynamic impact strength are separately increased by 39.4% and 56.1%, respectively. Comparing a STF concentration increasing from 30 wt% to 35 wt%, as well as from 35 wt% to 40 wt%, the former contributes to a greater energy absorption and better mechanical performance, whereas the latter reaches the critical value of SiO_2_ nanoparticles with which the STF can be loaded, which contributes to a less significant improvement in the mechanical properties of STF composite nonwoven fabrics. In terms of application, this can be mainly used in car interior decoration, personal protective equipment lining, etc.

## Figures and Tables

**Table 1:** Manufacturing parameters of nonwoven fabrics.

Specifications	
Fire-retardant polyester fibers (wt%)	50
Nomex recycle woven selvedge (wt%)	20
Low melting polyester fibers (wt%)	30
Needle punching times (needle/min)	200
Needle punching depth (cm)	1.75
Basis weight (g/m^2^)	300
Hot pressing temperature (°C)	140

**Table 2:** Summary of tensile strength and tearing strength data.

STF Concentration (wt%)	Tensile Strength (N)		Tearing Strength (N)	
	MD	CD	MD	CD
None	67.9 ± 6.0	153.0 ± 8.8	104.3 ± 4.7	136.1 ± 11.9
30	121.3 ± 5.3	280.1 ± 9.5	112.2 ± 5.7	180.4 ± 12.8
35	128.6 ± 3.2	304.2 ± 9.7	122.6 ± 5.5	215.0 ± 6.7
40	137.5 ± 5.8	330.4 ± 7.2	141.3 ± 8.1	246.3 ± 6.8

**Table 3:** Summary of bursting strength and dynamic impact data.

STF Concentration (wt%)	Bursting Strength (N)	Dynamic Impact (N)
None	512.0 ± 10.6	207.7 ± 12.5
30	557.7 ± 9.8	249.2 ± 13.5
35	662.6 ± 12.1	315.6 ± 14.1
40	713.8 ± 11.8	324.3 ± 4.7

## Data Availability

Not applicable.
